# The influence of mitochondrial biological function on sudden sensorineural hearing loss: Exploring potential mechanisms and associations through Mendelian randomization analysis

**DOI:** 10.1097/MD.0000000000045151

**Published:** 2025-11-07

**Authors:** Jialei Chen, Chang Hao, Xu Tang, Hongbing Yao, Shuping Su

**Affiliations:** a Department of Otolaryngology Head and Neck Surgery, Children’s Hospital of Chongqing Medical University, National Clinical Research Center for Child Health and Disorders, Ministry of Education Key Laboratory of Child Development and Disorders, Chongqing Key Laboratory of Child Neurodevelopment and Cognitive Disorders, Chongqing, China; b Department of Anesthesiology, Children’s Hospital of Chongqing Medical University, National Clinical Research Center for Child Health and Disorders, Ministry of Education Key Laboratory of Child Development and Disorders, Chongqing Key Laboratory of Child Neurodevelopment and Cognitive Disorders, Chongqing, China.

**Keywords:** GWAS, Mendelian randomization, mitochondrial biological function, risk factors, sudden sensorineural hearing loss

## Abstract

Mitochondrial dysfunction, particularly involving energy metabolism, oxidative stress, and structural integrity, is recognized as a critical factor in the pathogenesis of hearing loss. However, evidence regarding a potential association between mitochondrial function and sudden sensorineural hearing loss (SSNHL) remains limited. This study aims to explore the potential causal relationship between mitochondrial biological function and SSNHL using Mendelian randomization (MR) analysis. We utilized genome-wide association study data on SSNHL from the FinnGen database, along with mitochondria-related genome-wide association study summary statistics from previously published studies. Inverse variance weighting served as the primary MR method, using genetic instruments associated with mitochondrial biological function to estimate their effects on SSNHL risk. Sensitivity analyses—including MR-Egger regression, MR-PRESSO, and leave-one-out analysis—were conducted to assess the robustness of the findings and to exclude potential biases. Three mitochondrial proteins were found to exhibit potential causal effects on SSNHL. MUL1 was identified as a potential risk factor, whereas HINT2 and GRPEL1 appeared to serve as potential protective factors. This study provides evidence supporting a potential causal role of mitochondrial biological function in SSNHL. The findings offer novel insights into the mechanistic underpinnings of SSNHL and emphasize the importance of mitochondrial pathways in its pathophysiology. These results may encourage otolaryngologists to consider targeting mitochondrial energy metabolism in the development of more effective clinical prevention and treatment strategies for SSNHL.

## 1. Introduction

Sudden sensorineural hearing loss (SSNHL) is clinically defined as an acute-onset, idiopathic hearing impairment of ≥ 30 dB across at least 3 contiguous frequencies occurring within a 72-hour window.^[1]^ As an otologic emergency affecting cochleovestibular function, SSNHL manifests as partial or complete auditory deprivation. Early intervention within the critical therapeutic window (≤48 hours post-onset) significantly improves the prognosis for hearing recovery and reduces the risk of long-term disability.^[2]^ Current epidemiological data estimates indicate that the incidence of SSNHL ranges from 5 to 20 cases per 100,000 person-years.^[3]^ Alarmingly, epidemiological data reveal a progressive increase in SSNHL prevalence worldwide. Hearing loss is the fourth leading cause of disability worldwide, with approximately 466 million people affected globally.^[4]^ By 2030, this number is expected to increase to nearly 630 million individuals. Furthermore, as the population continues to age, it is projected that by 2050, over 900 million people will experience some degree of hearing loss.^[5]^ Autoimmune dysregulation,^[6]^ inflammatory processes,^[7]^ and viral infections^[8]^ represent proposed etiological factors for SSNHL, but its precise pathophysiology remains incompletely elucidated. Therefore, for otolaryngologists, understanding and accurately identifying the etiology of SSNHL is essential for its effective treatment and prevention.

Mitochondria, often referred to as the powerhouses of the cell, are responsible for generating adenosine triphosphate (ATP) and play a crucial role in the regulation of apoptosis, cellular metabolism, calcium homeostasis, and redox balance.^[9]^ Recent extensive studies have indicated that mitochondrial dysfunction is associated with various types of hearing loss.^[10]^ Age-related hearing loss is a progressive decline in hearing that occurs with aging. Research has indicated that age-related hearing loss is associated with mitochondrial DNA damage, excessive production of reactive oxygen species (ROS), and a decline in antioxidant function. The accumulation of ROS can lead to damage in inner ear cells, particularly hair cells and spiral ganglion cells, resulting in hearing impairment.^[11]^ Additionally, noise exposure is also a significant factor contributing to hearing loss. The generation of ROS induced by noise can damage mtDNA, reduce mtDNA content, decrease mitochondrial gene expression and ATP levels, and induce apoptosis in outer hair cells and inner hair cells, ultimately leading to hearing impairment.^[12]^ Unfortunately, to our knowledge, there is limited evidence investigating the association between SSNHL and mitochondrial dysfunction. Only a few studies have explored the potential links between mitochondrial genetic polymorphisms and susceptibility to SSNHL. Although the mechanisms of mitochondrial dysfunction have been partially elucidated in the context of specific types of hearing loss, the exact causal relationship between mitochondrial dysfunction and the pathogenesis of SSNHL remains unclear.

Mendelian randomization (MR) analysis is a method that uses genetic variation as instrumental variables (IVs) to infer causal relationships between modifiable risk factors and disease outcomes from observational data.^[13]^ This approach draws on the principle of random allocation used in randomized controlled trials, as the random distribution of genotypes occurs prior to fertilization and is unaffected by individual behavior or environmental factors. Consequently, MR can help mitigate the confounding effects and reverse causation, providing more reliable evidence. MR has been widely applied in causal inference for various diseases, including cardiovascular diseases,^[14]^ type 2 diabetes,^[15]^ and cancer.^[16]^ Through this method, researchers can more accurately assess the causal effects of certain exposures (such as blood pressure, lipid levels, and smoking) on disease outcomes. Therefore, in this study, we aim to comprehensively evaluate the role of mitochondrial biological function in SSNHL using genome-wide association study (GWAS) data to gain new insights.

## 2. Materials and methods

### 2.1. Study design

**Figure 1.**
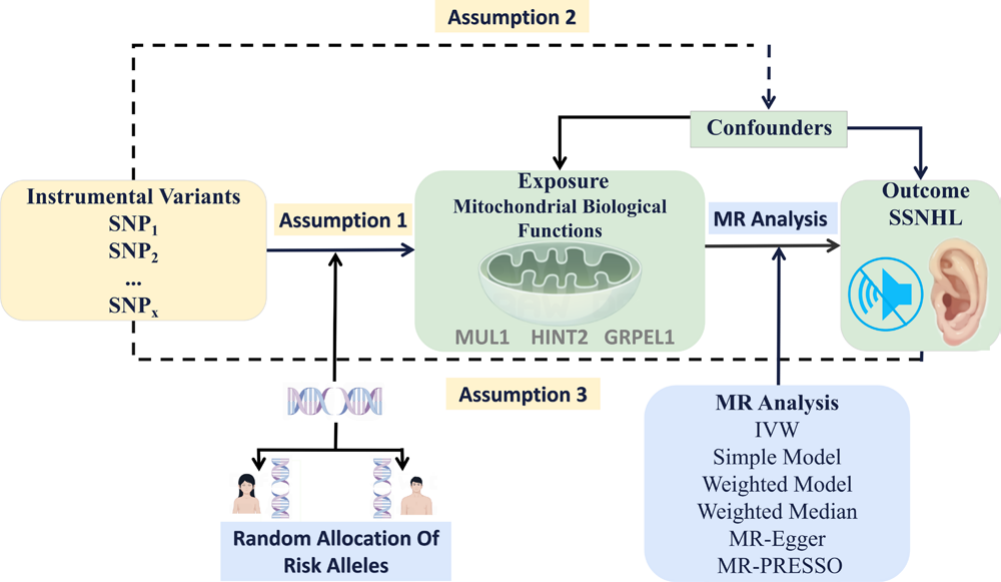
Overview of the MR analysis framework used to assess the causal relationship between mitochondrial biological function and SSNHL. SSNHL = sudden sensorineural hearing loss.

Figure 1 illustrates the framework utilized in this study. Essentially, we employed GWAS data related to mitochondrial biological function as the exposure variable and SSNHL datasets as the outcome variable for a 2-sample MR analysis. Based on stringent inclusion and exclusion criteria, single nucleotide polymorphisms (SNP) closely associated with mitochondrial biological function were selected as IVs. To minimize population stratification bias, both the exposure and outcome cohorts were restricted to individuals of European ancestry. A robust MR design is based on the following 3 fundamental assumptions^[17]^: Relevance assumption: the selected IVs (e.g., SNP) must be significantly associated with the exposure of interest. This means that the IVs should effectively predict the exposure, ensuring that there is a strong correlation between the IVs and the exposure. Independence assumption: The IVs must be independent of any confounding factors that could influence the outcome variable. In other words, the relationship between the genetic variant and the outcome should be solely mediated by the exposure, with no association between the IVs and other potential confounders that could bias the results. Exclusion assumption: the IVs should influence the outcome exclusively through the exposure. This means that the IVs should not have a direct effect on the outcome variable or affect it through other pathways (e.g., environmental or lifestyle factors). MR studies utilize existing genetic data from GWAS for analysis, thereby eliminating the need for additional ethical approvals.

### 2.2. Data sources

The mitochondrial biological function and its associated GWAS data were sourced from the following databases: https://gwas.mrcieu.ac.uk/ and https://www.ebi.ac.uk/gwas/. Genetic data associated with SSNHL were derived from the publicly accessible FinnGen database (Phenocode: H8_HL_IDIOP), comprising 212,544 participants (15,952 cases vs 196,592 controls). Case ascertainment utilized the International Classification of Diseases (ICD-10) code H91.2 through linkage with the Finnish Hospital Discharge Register, ensuring diagnostic standardization across all SSNHL patients.

### 2.3. Instrumental variables selection

Firstly, to satisfy the core assumption of a strong correlation between the IVs and the exposure factors, our study employed a significance threshold of *P* < 5 × 10^‐6^ for the genome-wide exposure.^[18]^ Empirical evidence from prior MR studies indicates that even using a screening threshold of *P* < 5 × 10⁻^6^, the likelihood of weak IVs introducing bias in the MR analysis remains low when performing linear regression on each genetic variant related to the exposure variable.^[19]^ Secondly, we established thresholds of *R*^2^ < 0.001 and kb > 10 Mb to exclude SNP that exhibited linkage disequilibrium during the IVs selection process, ensuring that the likelihood of alleles from 2 or more loci co-occurring on a single chromosome is greater than that of random occurrence.^[20]^ We also eliminated palindromic SNP to ensure a consistent effect of the alleles on both the exposure and the outcome. The *F*-statistic is used to assess the bias caused by IVs. To reduce estimation bias caused by IVs, the equation *F* = (*R*^2^ × (n ‐ 2))/(1 ‐ *R*^2^) is employed to evaluate the strength of the instruments and the relationship between exposure and outcome. The estimated *R*^2^ for the IVs is calculated using the formula 2EAF(1 ‐ EAF) * β^2^, where EAF denotes the frequency of the effect allele, and β represents the estimated genetic effect on the exposure factors.^[21]^ Table S1, Supplemental Digital Content, https://links.lww.com/MD/Q385 provides specific SNP information along with the corresponding *R*^2^ and *F* statistics. A significant correlation is considered to exist when *F* ≥ 10. The PhenoScanner database (https://www.repository.cam.ac.uk/items/31e4df31-982b-452e-baf8-83ce942b1c0a) was used to exclude all known phenotypes associated with any genetic instruments considered in our analysis.^[22]^

### 2.4. Mendelian randomization analysis

Researchers employed the inverse variance weighting (IVW) method as the primary analysis to assess the causal relationship between mitochondrial biological function (exposure factor) and SSNHL (outcome).^[23]^ Additionally, we utilized the weighted median method, simple mode method, weighted mode method, and MR-Egger method to rigorously examine the causal relationship, thereby enhancing the reliability and accuracy of our study findings.^[24]^

### 2.5. Sensitivity analysis

To determine the presence of pleiotropy in the IVs and its potential impact on the outcomes, we conducted sensitivity analyses. Cochran *Q* test was employed to assess SNP heterogeneity, with a *P*-value less than .05 indicating the presence of heterogeneity. In the MR-Egger regression effect model, if the intercept is close to zero or the significance level exceeds 0.05, it indicates that pleiotropy does not have an impact on the IVs.^[24]^ The MR-PRESSO method systematically evaluates the impact of pleiotropy through a global test. Furthermore, a leave-one-out analysis was performed to assess whether the bias in MR estimates was driven by any single SNP.^[25]^ In addition, we plotted a funnel plot to evaluate the symmetry of the SNP and assess the reliability of the results.

### 2.6. Statistical analysis

All statistical analyses were conducted using the R package “TwoSampleMR” (version 0.5.6; MRC Integrated Epidemiology Unit [MRC IEU], University of Bristol, Bristol, United Kingdom) in R (version 4.2.1). For more detailed instructions, please refer to the following link: https://mrcieu.github.io/TwoSampleMR/articles/gwas2020.html. Online resources were used to calculate statistical power for the MR investigation (https://sb452.shinyapps.io/power/). Statistical significance for the estimated causal effects of the exposure was determined when the *P*-value was less than .05.

## 3. Results

### 3.1. Dataset screening

**Figure 2.**
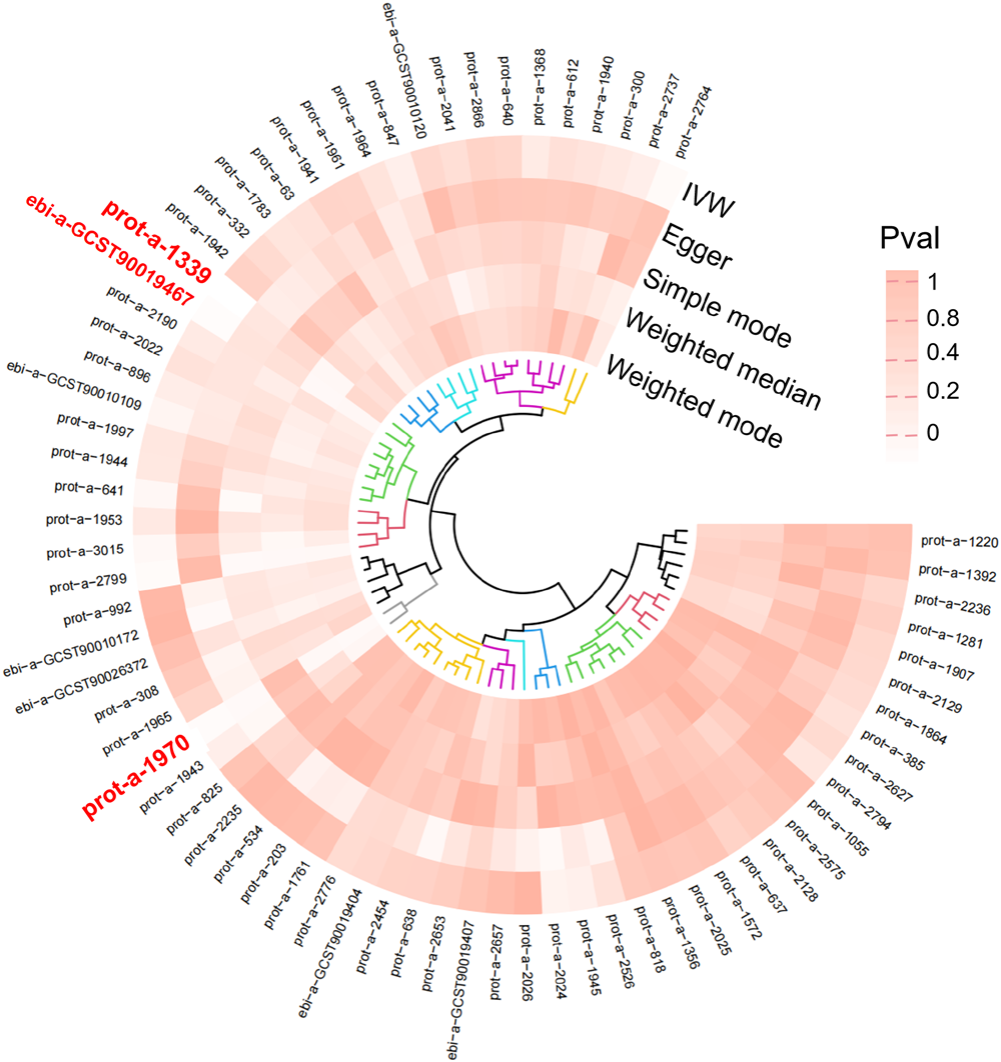
SNPs associated with mitochondrial biological function and SSNHL. SNP = single nucleotide polymorphisms, SSNHL = sudden sensorineural hearing loss.

As shown in Figure 2, we identified 3 datasets (prot-a-1339, histidine triad nucleotide-binding protein 2, HINT2; prot-a-1970, mitochondrial ubiquitin ligase activator of NFKB 1, MUL1; ebi-a-GCST90019467, GrpE protein homolog 1, GRPEL1) with a potential causal relationship to SSNHL from a total of 71 datasets related to mitochondrial biological function.

### 3.2. MR analysis results

**Figure 3.**
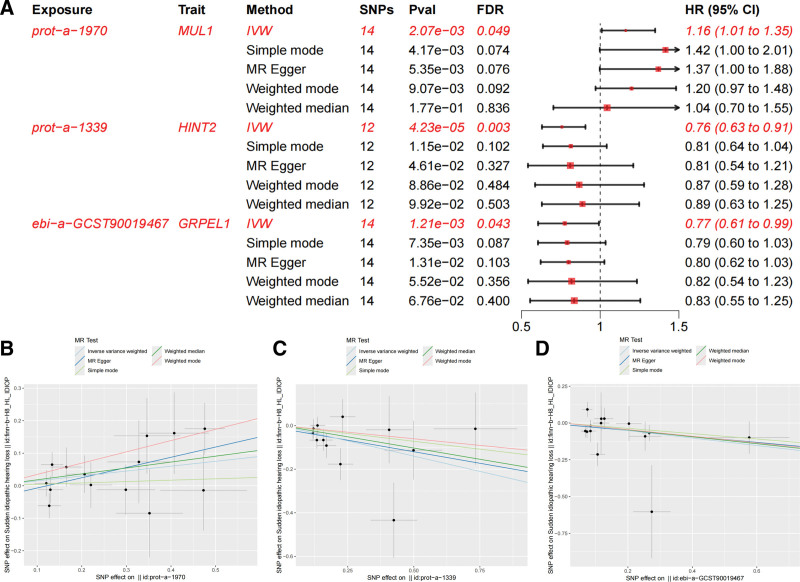
MR analysis of the causal relationship between mitochondrial biological function and SSNHL. (A) Causal relationship between 3 identified mitochondrial biological function and SSNHL. FDR represent for the *P*-value adjusted by the false discovery rate. (B–D) Scatter plots illustrate the causal relationship between prot-a-1970, prot-a-1339 and ebi-a-GCST90019467 and SSNHL. Each point represents the association of a single genetic variant (SNP) with the exposure (*X*-axis) and with the outcome (*Y*-axis). FDR = false discovery rate, SNP = single nucleotide polymorphisms, SSNHL = sudden sensorineural hearing loss.

MR analysis utilizes genetic variations to determine whether the observed associations between risk factors and outcomes are consistent with potential causal relationships. All IVs demonstrated strong instrument validity (mean *F*-statistic = 296.99, range: 18.99–126.84), effectively mitigating weak instrument bias. After assessing and removing SNPs associated with confounding factors, the overall results of the 2-sample MR analysis of mitochondrial biological function and SSNHL were illustrated in Figure 3A. To provide a more intuitive representation of the MR analysis results, we also plotted a scatter plot. For MUL1, a total of 14 SNPs were included in the analysis. In the IVW analysis, HINT2 was identified as a risk factor for the onset of SSNHL (OR = 1.16, 95% CI = 1.01–1.35, *P* = 2.07 × 10^−3^, false discovery rate (FDR) corrected *P* = .049). Additionally, HINT2 (OR = 0.76, 95% CI = 0.63–0.91, *P* = 4.23 × 10^−5^, FDR corrected *P* = .003) and GRPEL1 (OR = 0.77, 95% CI = 0.61–0.99, *P* = 1.21 × 10^−3^, FDR corrected *P* = .043) each had 12 and 14 SNPs, respectively, included in the IVW analysis and were both identified as protective factors for SSNHL. Similarly, the weighted median method, simple mode method, weighted mode method, and MR-Egger method also demonstrated the same trend, although the associations did not reach statistical significance (Fig. 3B–D). In addition, we discuss the statistical power of this study. For MUL1, based on a sample size of 212,544, a case-control ratio of 0.075, a genetic variant variance of 0.0095, and an odds ratio of 1.16, we calculated the statistical power for this result, which was 100%. Regarding HINT2, using a sample size of 212,544, a case-control ratio of 0.075, a genetic variant variance of 0.0075, and an odds ratio of 0.76, the statistical power for this result was computed to be 100%. In the case of GRPEL1, with a sample size of 212,544, a case-control ratio of 0.075, a genetic variant variance of 0.0028, and an odds ratio of 0.77, the statistical power for this result was calculated to be 99.99%.

### 3.3. Sensitivity analysis

**Figure 4.**
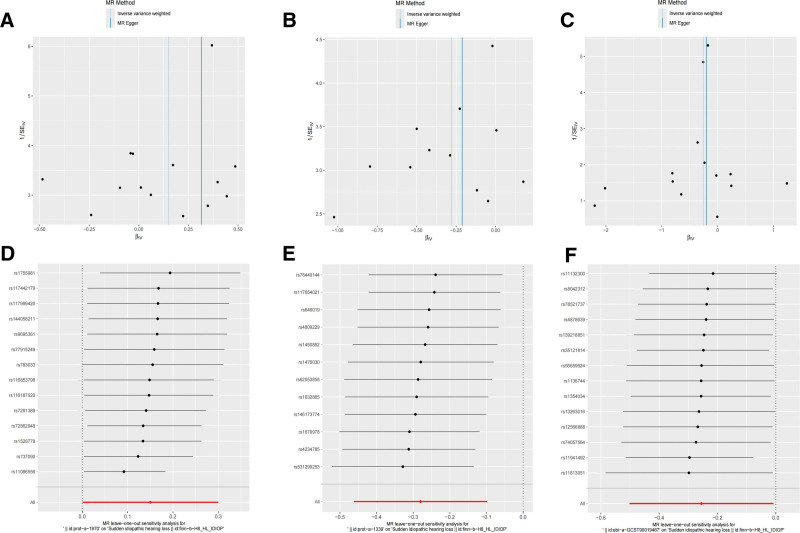
Sensitivity analysis between 3 identified mitochondrial biological function and SSNHL. (A–C) Funnel plots illustrating the causal relationship between prot-a-1970, prot-a-1339 and ebi-a-GCST90019467 and SSNHL. The funnel plot displays the individual effect estimates of each SNP on the *X*-axis against their precision (typically measured as 1/standard error) on the *Y*-axis. (D–F) Leave-one-out plots depicting the causal relationship between prot-a-1970, prot-a-1339 and ebi-a-GCST90019467 and SSNHL. The points in the figure represent individual SNPs, with the *X*-axis indicating the SNP that were removed and the *Y*-axis displaying the combined effect estimates after the removal of each SNP. SNP = single nucleotide polymorphisms, SSNHL = sudden sensorineural hearing loss.

The null hypothesis of the MR-Egger intercept test asserts that “there is no directional pleiotropy.” A *P*-value greater than .05 signifies insufficient statistical evidence to reject this hypothesis. Similarly, the MR-PRESSO global test’s null hypothesis asserts that “none of the instrumental variables exhibit pleiotropy.” A *P*-value above .05 indicates that the observed pleiotropy evidence is not significant compared to the null model, suggesting that no overall pleiotropy was detected. As shown in Table 1, both the MR-Egger intercept and MR-PRESSO tests did not indicate significant pleiotropy (*P* > .05). Similarly, neither the MR-Egger nor the IVW methods showed significant heterogeneity in the Cochran *Q* test (*P* > .05). The funnel plot displays the individual effect estimates of each SNP on the *X*-axis against their precision (typically measured as 1/standard error) on the *Y*-axis. In the absence of heterogeneity and pleiotropy, the points should be roughly symmetrically distributed around the overall combined estimate (IVW or MR-Egger) on either side. Our funnel plot demonstrated a symmetrical distribution of SNPs, highlighting the relative stability of the results (Fig. 4A–C). Leave-one-out sensitivity analysis indicated that our overall causal estimates are robust (Fig. 4D, E). After sequentially removing any single SNP, the causal effect estimates derived from the remaining set of SNPs closely align with the global estimate (represented by the black line in the figure), and there is substantial overlap in the confidence intervals. This suggests that our conclusions are not driven by any single influential or potentially pleiotropic outlier SNP. While our sensitivity analysis results provide some preliminary evidence for the exclusion of major directional pleiotropy, we cannot assert that residual pleiotropy has been completely eliminated. Residual pleiotropy, particularly subtle or symmetrical forms, remains a potential threat that cannot be entirely ruled out, and this represents an inherent limitation of Mendelian randomization studies.

**Table 1:** Pleiotropy and heterogeneity test of mitochondrial biological function genetic IVs in GWAS for SSNHL.

GWAS-ID	Type	MR-Egger intercept test		Cochran *Q* test				MR-PRESSO	
		Egger_intercept	*P*_val_	*Q* (Ivw)	*Q*_Pval_	*Q* (MR-Egger)	*Q*_Pval_	Outlier	*P*_val_
prot-a-1970	SSNHL	‐0.038	0.27	12.29	0.50	10.96	0.53	14.80	0.50
prot-a-1339	SSNHL	‐0.014	0.71	11.85	0.37	11.69	0.30	13.97	0.44
ebi-a-GCST90019467	SSNHL	‐0.011	0.74	17.00	0.19	16.85	0.15	18.07	0.31

GWAS = genome-wide association study, IVs = instrumental variables, SSNHL = sudden sensorineural hearing loss.

## 4. Discussion

Hearing loss is a prevalent global health issue affecting millions of people worldwide. In recent years, growing evidence has indicated that mitochondrial dysfunction plays a critical role in the onset and progression of hearing loss.^[26]^ However, direct evidence linking mitochondrial function to SSNHL remains scarce, largely due to limitations in animal models and an underdeveloped theoretical framework. This study is the first to systematically investigate the potential causal relationship between mitochondrial biological function and SSNHL using a 2-sample MR approach, thereby addressing a significant gap in the current literature. Mitochondria serve as the primary organelles for cellular energy production (ATP) and are essential in regulating cellular metabolism, apoptosis, calcium homeostasis, and redox balance.^[9]^ The blood supply to the cochlea depends predominantly on the labyrinthine artery, which has no collateral circulation. Meanwhile, hair cells—responsible for transducing auditory and vestibular signals—exhibit high metabolic activity and possess a dense concentration of mitochondria, making them heavily reliant on oxygen and energy metabolism. These cells demand substantial amounts of ATP to sustain normal function. Following injury, their energy requirements escalate further, increasing the vulnerability of the auditory system to mitochondrial dysfunction.^[27]^

SSNHL is a distinct form of sensorineural hearing loss that arises without any identifiable cause. In this study, HINT2 and GRPEL1 were identified as protective factors against SSNHL. HINT2 is a mitochondrial enzyme that functions primarily as a nucleotide hydrolase and transferase. It plays a vital role in regulating mitochondrial function, energy metabolism, calcium homeostasis, and apoptosis.^[28]^ Studies have demonstrated that HINT2 knockout mice exhibit deficits in mitochondrial bioenergetics, including reduced ATP production and lower mitochondrial membrane potential.^[29]^ Furthermore, HINT2 helps preserve mitochondrial function by inhibiting the activation of the mitochondrial calcium uniporter complex, thereby attenuating damage caused by microvascular ischemia–reperfusion.^[30]^ Li et al reported that high expression of HINT2 can effectively reduce oxidative stress in tissues.^[31]^ Given that ischemia–reperfusion injury and oxidative stress are recognized as key mechanisms in the pathogenesis of SSNHL,^[32,33]^ the upregulation of HINT2 may help maintain stable energy metabolism in inner ear mitochondria and reduce oxidative stress following injury. This could represent 1 mechanism through which HINT2 acts as a protective factor in SSNHL. GRPEL1 plays a pivotal role in maintaining mitochondrial protein stability. It functions in concert with mitochondrial heat shock protein 70 (mtHsp70) to facilitate protein import into the mitochondrial matrix, stabilize protein complexes, and promote proper protein folding. Its core activity is closely tied to the adenosine diphosphate–ATP exchange process mediated by mtHSP70.^[34]^ Studies have demonstrated that tissue-specific knockout of GRPEL1 in mouse skeletal muscle leads to muscle atrophy, suppresses oxidative phosphorylation and mitochondrial fatty acid oxidation, and induces pronounced proteotoxic stress.^[35]^ These findings establish GRPEL1 as a key regulator of tissue-specific mitochondrial function and metabolic homeostasis. GRPEL1 also contributes significantly to the mechanisms of neurodegenerative diseases. Research by Ma et al indicates that GRPEL1 participates in regulating the mitochondrial unfolded protein response (UPRmt) following experimental subarachnoid hemorrhage. Upregulation of GRPEL1 enhances its interaction with mtHSP70—a critical process for repairing mitochondrial damage—and supports functional recovery in impaired neurons.^[36]^ Given that virus-induced neuronal dysfunction has been proposed as one of the core pathological mechanisms in SSNHL,^[37,38]^ the ability of GRPEL1 to promote neuronal recovery may underlie its role as a protective factor against SSNHL. MUL1, a ubiquitin ligase localized to the mitochondrial outer membrane, regulates multiple pathophysiological processes including apoptosis, mitophagy, mitochondrial dynamics, and innate immune responses.^[39]^ Recent studies in rat models of ischemic stroke have shown that upregulation of MUL1 disrupts mitochondrial dynamics and exacerbates cerebral injury.^[40]^ Similarly, Wang et al reported increased MUL1 protein levels during myocardial ischemia/reperfusion injury in rats. Inhibition of MUL1 expression was found to ameliorate mitochondrial dysfunction, thereby reducing cellular apoptosis and necrosis.^[41]^ Notably, MUL1 exhibits a dual role in mitochondrial regulation: both overexpression and deficiency can disrupt mitochondrial homeostasis.^[42]^ One compelling study demonstrated that knockout of the MUL1 gene exacerbates mitochondrial damage and induces neuronal degeneration.^[43]^ Furthermore, Wu et al observed significantly reduced MUL1 expression in cases of age-related hearing loss.^[44]^ Taken together, abnormal expression of MUL1 may represent a risk factor for the development of SSNHL.

To our knowledge, this study provides the first systematic analysis of the potential causal relationship between mitochondrial biological function and SSNHL. Our results identify 3 mitochondrial proteins with potential causal roles in SSNHL pathogenesis: MUL1 as a risk factor, and HINT2 and GRPEL1 as protective factors. These results provide genetic evidence supporting a potential causal role of mitochondrial dysfunction in SSNHL, offering novel insights into its underlying mechanisms. It is important to acknowledge several limitations of this study. Firstly, the genomic data were derived from multiple sources, which may introduce heterogeneity. Although statistical tests did not detect significant heterogeneity, further validation in more homogeneous cohorts is warranted. Secondly, as the analysis primarily included individuals of European ancestry, the generalizability of our results to other populations remains limited. Future studies should incorporate more diverse ethnic groups to confirm these associations. Finally, while our study identifies potential causal relationships, the precise biological mechanisms remain unclear. Functional experiments are essential to elucidate the specific pathways through which these mitochondrial proteins influence SSNHL.

## Acknowledgments

The authors thank the FinnGen study in our analysis for providing a publicly available GWAS dataset.

## Author contributions

**Conceptualization:** Jialei Chen, Chang Hao, Xu Tang, Hongbing Yao, Shuping Su.

**Data curation:** Jialei Chen, Chang Hao, Xu Tang, Shuping Su.

**Funding acquisition:** Shuping Su.

**Investigation:** Jialei Chen, Chang Hao, Xu Tang, Hongbing Yao, Shuping Su.

**Project administration:** Shuping Su.

**Writing – original draft:** Jialei Chen, Chang Hao, Xu Tang, Hongbing Yao, Shuping Su.

**Writing – review & editing:** Jialei Chen, Chang Hao, Xu Tang, Hongbing Yao, Shuping Su.

## Supplementary Material


